# Voltage-Frequency Domain Optimization for Energy-Neutral Wearable Health Devices

**DOI:** 10.3390/s20185255

**Published:** 2020-09-14

**Authors:** Yigit Tuncel, Sizhe An, Ganapati Bhat, Naga Raja, Hyung Gyu Lee, Umit Ogras

**Affiliations:** 1 Department of Electrical and Computer Engineering, University of Wisconsin-Madison, Madison, WI 53706, USA; san49@wisc.edu (S.A.); uogras@wisc.edu (U.O.); 2 School of Electrical Engineering and Computer Science, Washington State University, Pullman, WA 99164, USA; ganapati.bhat@wsu.edu; 3 School of Electrical, Computer and Energy Engineering, Arizona State University, Tempe, AZ 85287, USA; nraja1@asu.edu; 4 School of Computer and Communication Engineering, Daegu University, Gyeongsan 38453, Korea; hglee@daegu.ac.kr

**Keywords:** voltage-frequency domains, optimization, wearable devices, low-power design, energy consumption

## Abstract

Wearable health and activity monitoring devices must minimize the battery charging and replacement requirements to be practical. Numerous design techniques, such as power gating and multiple voltage-frequency (VF) domains, can be used to optimize power consumption. However, circuit-level techniques alone cannot minimize energy consumption unless they exploit domain-specific knowledge. To this end, we propose a system-level framework that minimizes the energy consumption of wearable health and activity monitoring applications by combining domain-specific knowledge with low-power design techniques. The proposed technique finds the energy-optimal VF domain partitioning and the corresponding VF assignments to each partition. We evaluate this framework with experiments on two activity monitoring and one electrocardiogram applications. Our approach decreases the energy consumption by 33–58% when compared to baseline designs. It also achieves 20–46% more savings compared to a state-of-the-art approach.

## 1. Introduction

Advances in wearable devices have led to a variety of health and activity monitoring applications, such as pacemakers and electrocardiogram (ECG) trackers [1]. Wearable solutions allow for the monitoring of everyday routines of users in their home environment that can lead to a multitude of quality of life improvements, such as remote diagnostics and biofeedback, and even promote preventive medicine [2]. However, frequent charging requirements and privacy concerns prevent wearable healthcare devices from being a ubiquitous technology [3]. A recent survey demonstrates that nearly one-fourth of wearable device owners stop using the device, because charging is too frequent and inconvenient [4]. A considerable number of users also do not want their personal data uploaded to a remote device due to privacy concerns [3]. Therefore, there is a strong need for energy-neutral devices that perform local processing of the data. Energy-neutrality ensures that the energy consumption in a given period, such as a day, is equal to the energy harvested during the period. Consequently, achieving energy-neutrality in wearable devices will eliminate their charging requirements.

**Figure 1:**
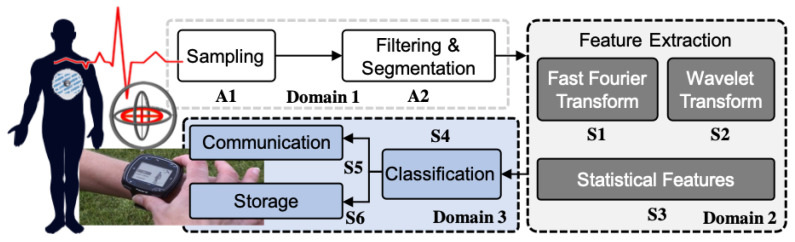
A sample activity monitoring application with three VF domains. Modules A1 and A2 continuously sample and process data, while the other modules become active only when a data frame is ready.

Health and activity monitoring applications consist of multiple processing pipelines that may not be active simultaneously. The front-end of the pipeline samples, filters, and stores data from multiple sensors, such as accelerometer and ECG electrodes, as illustrated in Figure 1. Because the speed of human body dynamics ranges from a few hundreds of milliseconds to seconds, a sequence of samples (i.e., a data frame) is needed to extract useful information from sensors. For example, 1–2 s of data is needed to perform human activity monitoring [5]. Therefore, sensors, analog front end, and preprocessing modules run continuously in order to construct data frames when the user is active. In contrast, processing modules do not need to operate until their inputs are available. For example, the feature extraction modules in Figure 1 can wait in a low-power sleep state until the data is segmented. Likewise, the classification module can wake up after feature extraction is complete. Because this pipeline behavior is broadly applicable to other health and activity monitoring applications, it can be utilized to minimize energy consumption. To this end, we leverage this domain-specific knowledge to optimally partition the modules into multiple voltage-frequency (VF) domains.

This paper presents a system-level optimization framework to help achieve energy-neutrality in wearable devices for health and activity monitoring applications. One of the primary challenges for achieving energy-neutral devices is that the power consumption of health monitoring applications is higher than the harvesting potential of ambient sources. We can solve this by increasing the harvested energy while decreasing the energy consumption of the application. Because the energy harvesting potential is limited, we must reduce the energy consumption of the target application through low-power design practices, such as clock and power gating. To this end, we focus on minimizing the energy consumption of health monitoring applications by optimizing the VF domain partitioning. We achieve this using two complementary techniques. The first technique finds the energy-optimal voltage-frequency assignments to each domain in a given arbitrary VF domain partition for a target application. The second technique is a VF domain partitioning algorithm that explores the design space by evaluating different partitioning configurations using the first technique. Comparisons to exhaustive search reveal that the energy consumption results with the partitions obtained by the proposed framework are within 3% of the minimum achievable energy.

The proposed framework is evaluated experimentally using human activity monitoring and ECG applications. We designed and implemented the activity monitoring application in TSMC 65 nm LP process in prior studies [6,7]. For the current work, we first synthesize the baseline design to characterize the switching capacitance, leakage current, and the number of active cycles of each module and use these inputs along with the application pipeline to determine the optimal VF domain configuration. Finally, we implement the optimal configuration to validate the proposed framework. Our detailed evaluation using Synopsys PrimeTime and user activity data shows that we achieve 1.5 μJ energy consumption per activity, which is 45% lower than a baseline with two VF domains. Moreover, the proposed technique achieves 12% lower energy consumption than a state of the art method [8]. Similarly, we achieve approximately 35% energy savings on an ECG application when compared to the literature [1]. Hence, we argue that these applications can be sustained by either a 1 cm^2^ indoor solar harvester that generates 100 μJ every second at 0.1 mW/cm^2^ [9], a small (0.1 cm^3^) electromagnetic human motion harvester that generates 70 μJ at 0.7 mW/cm^3^ [10], or a flexible piezoelectric based energy harvester that generates 7.8 μJ per step during walking [11] to achieve energy-neutrality.

The major contributions of this work are:

- A technique that finds the energy-optimal voltage and frequency levels for a given design and VF domain partition;
- An exact algorithm and an efficient heuristic to find the optimum VF domain configuration; and,
- Experimental evaluation on three applications: two activity monitoring applications implemented using TSMC 65 nm LP technology and a low-power ECG application from the literature.

In the rest, Section 2 reviews the previous work and highlights our contributions. Section 3 introduces the application and hardware models. Section 4 presents the optimal VF domain assignment techniques. Section 5 presents the experimental validation and Section 6 concludes the paper.

## 2. Related Work

Low-power design techniques have been extensively studied due to their applications in energy-constrained devices. They can be broadly classified as circuit- or system-level optimizations [12,13]. Circuit-level approaches analyze the logic in each module and insert power/clock gating blocks to achieve power savings. For example, the impact of voltage distribution and DC-DC converter placement on power consumption is analyzed in [12]. Blutman et al. [13] explore subblock-level partitioning for stacked power domain designs. Similarly, the approach in [14] analyzes the high-level description of a design and generates granular power domains. While circuit-level power optimizations are crucial, in many cases the designers do not have control over the details of individual sub-blocks. Therefore, recent research has also explored system-level techniques for low-power optimizations.

These techniques consider optimization for each computation module and assign the VF for these modules. For instance, Wang et al. [15] use an evolutionary algorithm to obtain a VF partitioning of modules in a voice-over LTE application. The work in [8] proposes an iterative algorithm to perform VF partitioning for network-on-chip (NoC) architectures. The approach starts with an individual VF domain for each NoC tile and progressively merges neighboring domains to find the partitioning with minimum energy. However, this approach does not exploit inherent pipelining in wearable applications since its focus is high-end systems. Furthermore, it considers merging only neighboring tiles, while our approach considers all possible options when merging domains. Finally, unlike previous approaches, we exploit application-specific information to obtain additional savings, as discussed in Section 5.3.

Application-specific power gating has been successfully used in [16,17]; however, these approaches rely on manual optimization that is not practical for large designs. In contrast, our proposed framework utilizes the inherent characteristics of the target application to generate the optimal VF domain partitioning and VF assignments. Specifically, we utilize application-specific knowledge, such as dependencies of tasks and the number of active clock cycles per module, in order to minimize the energy per activity in health monitoring applications. Using the proposed efficient algorithm, we find the configuration that provides minimum energy per activity.

## 3. Overview and Problem Formulation

### 3.1. Application Model

We consider health and activity monitoring applications that are similar to the example shown in Figure 1. These applications collect and analyze physiological data in realtime. They first construct a data frame by sampling the sensors for a duration of time, called an *activity window* ($t_{a}$). For instance, at least 1–2 s of gyroscope and accelerometer readings are needed to perform accurate human activity monitoring [5]. Once a sufficient number of data samples are collected and preprocessed (e.g., filtered as in Figure 1), a new data frame of length $t_{a}$ becomes ready for processing. This frame is then processed by the modules that perform the final analysis, such as feature extraction and classification. Because of this characteristic of activity monitoring applications, the modules that process a complete frame need to be active only when a new frame is ready. Using this insight, the hardware modules are divided into two broad categories: always-on and sporadic.

**Always-On Modules:** the modules that operate on every new data sample are referred to as *always-on*, as they stay active throughout the activity window $t_{a}$. This category includes the modules that interface the analog front-end and digital processing, such as filters and buffers (Domain 1 in Figure 1).

**Sporadic Modules:** the modules that run on a completed data frame are referred to as *sporadic*, since they become active only after their inputs are available and return to an idle state after producing an output. All of the modules that are part of the processing pipeline fall into this category.

### 3.2. Problem Formulation

Our goal is to minimize the energy consumption of health and activity monitoring applications, such that energy harvesting solutions can more easily sustain them. We denote the set of always-on modules as A and the set of sporadic modules as S. Let the dynamic capacitance, switching activity, and leakage current of module m∈{A,S} be $C_{m}$, $\alpha_{m}$, and $I_{m}$, respectively. Using the notation that is summarized in Table 1, we express the total energy consumption of always-on modules per activity window as a sum of its dynamic and leakage power consumption:

(1) $$E_{A}=\sum_{m\in A}\alpha_{m}C_{m}V^{2}\left(m\right)f\left(m\right)t_{a}+I_{m}V\left(m\right)t_{a}$$

where functions $V:\left\{A,S\right\}\to R^{+}$ and $f:\left\{A,S\right\}\to R^{+}$ give the voltage and frequency of module m∈{A,S}.

Unlike always-on modules, the active time of a sporadic module m∈S is given by the ratio of its active cycles ($n_{m}$) and frequency: $n_{m}/f\left(m\right)$. The total energy consumption of sporadic modules per activity window is:

(2) $$E_{S}=\sum_{m\in S}\alpha_{m}C_{m}V^{2}\left(m\right)n_{m}+I_{m}V\left(m\right)\frac{n_{m}}{f(m)}$$

**Figure 2:**
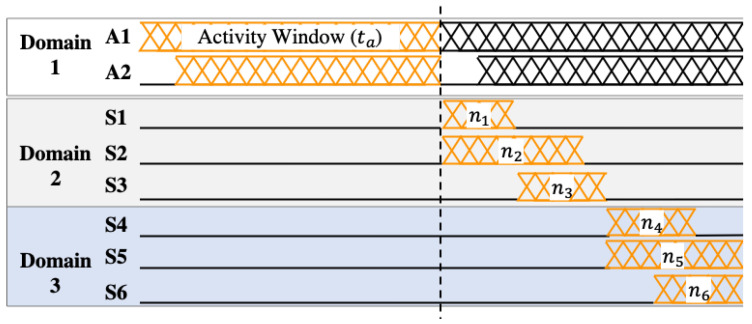
Timing information and activity window example.

The following problem formulation helps us find the number of VF domains, modules in each domain (as in Figure 1), and VF assignments that minimize the total energy consumption:

**Given:** A design with $\alpha_{m},C_{m},n_{m},$ and $I_{m}\;\forall m\in \left\{A,S\right\}$,
**Find:**
  (1) The number (*N*) and set of voltage-frequency domains $D=\{D_{1},D_{2},\ldots ,D_{N}\}$;
  (2) The mapping h:{A,S}→D of the modules to domains;
  (3) The voltage V(m) and frequency f(m) of each module m∈{A,S}.
**Such that:**
    (3) $$\begin{array}{cccc} & \min\;\;E_{total}=E_{A}+E_{S}+E_{overhead} & & \\ & f(m)=f(n)\;\mathrm{if}\;h(m)=h(n)\;\forall m,n\in \{A,S\} & \end{array}$$

where $E_{overhead}$ is the energy consumption overhead of introducing new voltage-frequency domains. The constraint in Equation (3) ensures that all of the modules in the same domain have the same voltage and frequency. For example, the inputs to the problem for the activity monitoring application in Figure 1 are the switching capacitance, leakage current, and activity factors after synthesizing the application in hardware. Besides this, the timing information of the sporadic modules (e.g., $n_{m}\;\forall m\in \left\{A,S\right\}$) is also provided to the problem. The timing information is obtained by simulating the hardware with real-world sensor data, as illustrated in Figure 2 for our activity monitoring application.

## 4. Optimal VF Domain Design

The proposed framework consists of two steps. First, we develop a technique that finds the energy-optimal voltage and frequency for a given VF domain partition (Section 4.1). Subsequently, we present an algorithm that uses this technique to find the VF domains D and the modules in them (Section 4.2). The proposed framework can also be used to optimize the energy-delay-product (EDP) if the designer prefers to emphasize performance. This can be achieved by expressing the EDP of always-on modules as $EDP_{A}=E_{A}t_{a}$ and that of sporadic modules as:

(4) $$EDP_{S}=\sum_{m\in S}\alpha_{m}C_{m}V^{2}\left(m\right)\frac{n_{m}^{2}}{f(m)}+I_{m}V\left(m\right)\frac{n_{m}^{2}}{f^{2}\left(m\right)}$$

### 4.1. Optimal Voltage and Frequency in a Domain

This section describes the methodology to compute the energy-optimum voltage and frequency for a design with given VF domains. For instance, three domains and the modules in them are given as inputs, as illustrated in Figure 1 and Figure 2. Our goal is to find the VF assignments that minimize $E_{total}$.

**Optimum Voltage-Frequency for*****Always-On Modules*****:** The energy consumption of always-on modules is given in Equation (1). The partial derivative of $E_{A}$ with respect to the frequency of a given module m∈A can be found as:

(5) $$\frac{\partial E_{A}}{\partial f(m)}=\alpha_{m}C_{m}V^{2}\left(m\right)t_{a}+2\alpha_{m}C_{m}V\left(m\right)\frac{dV(m)}{df(m)}f\left(m\right)t_{a}+I_{m}\frac{dV(m)}{df(m)}t_{a}\;\forall m\in A$$

The derivative of V(m) with respect to f(m) is greater than or equal to zero (dV(m)/df(m)≥0), since the minimum required supply voltage is a non-decreasing function of frequency [18].

Hence, the partial derivative of the always-on energy with respect to the operating frequency is positive. Consequently, always-on modules should operate at the minimum allowed operating frequency, which is limited by the fastest sensor sampling rate denoted by $F_{s}$ for the technology node. The processing time of module $m\in D_{i}$ must be less than or equal to $1/F_{s}$ in order to not miss any data point. If the processing takes $n_{m}$ cycles, the minimum frequency of module $m\in D_{i}$ can be found as:

(6) $$\frac{n_{m}}{f_{min}\left(m\right)}=\frac{1}{F_{s}}\Rightarrow f_{min}\left(m\right)=n_{m}F_{s}$$

The domain frequency must be greater than the minimum frequencies of the modules it contains. Hence, the optimum frequency for a given domain $D_{i}$ can be found as:

(7) $$f_{opt}=\max_{m\in D_{i}}f_{min}\left(m\right)$$

**Optimum Voltage-Frequency for*****Sporadic Modules*****:** Energy consumption of sporadic modules is given in Equation (2). Unlike the always-on modules, the partial derivative of $E_{S}$ with respect to frequency is not always positive. Furthermore, the processing deadline is a nontrivial function of the application pipeline and task dependencies. Hence, a closed-form solution for the sporadic modules is not guaranteed, unlike the always-on modules. Instead, the optimal frequency is found by solving the following nonlinear constrained optimization problem:

(8) $$\begin{array}{cccc} \; & \underset{V(m),f(m)}{\mathrm{minimize}}\; & \; & E_{S}=\sum_{m\in S}\alpha_{m}C_{m}V^{2}\left(m\right)n_{m}+I_{m}V\left(m\right)\frac{n_{m}}{f(m)}\\ \; & \mathrm{subject}\;\mathrm{to}\; & \; & t_{exe}\left(f\left(m\right),n_{m},D\right)\leq t_{max},m\in S \end{array}$$

where $t_{exe}\left(f\left(m\right),n_{m},D\right)$ gives the completion time of the whole pipeline as a function of the frequency assignments, active cycles of each module $m\in D_{i}$, and domain configuration.

The maximum time constraint $t_{max}$ is determined by the target application. For example, $t_{max}$ is in the order of seconds for human activity monitoring, while it is in the order of milliseconds for heartbeat monitoring. This information is available in the input design, as illustrated in Figure 2 and elaborated upon in Section 5.1.2. We solve this optimization problem in the domain partitioning algorithm using a standard solver, such as the *fmincon* function in MATLAB.

### 4.2. Optimum VF Domain Partitioning

The energy consumption of the design is a function of the partitioning of modules into domains. The greatest degree of freedom, hence the lowest application energy ($E_{A}+E_{S}$) is achieved if each module has its domain. However, this choice also incurs the highest energy consumption overhead due to larger number of VF domains. More specifically, each additional VF domain introduces energy overhead due to IR drop, regulator, level shifter, sleep transistors, and clock domain crossing. Because the VF domain overhead $E_{overhead}$ increases with the number of domains, the optimal solution can be at an intermediate point between a single VF domain and putting each module into a dedicated domain.

**Figure 3:**
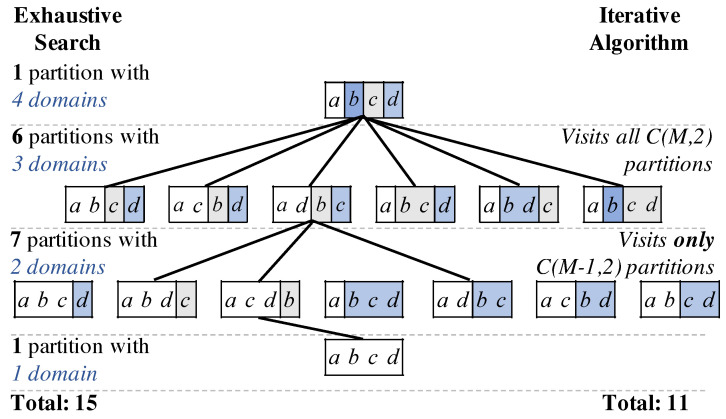
Illustration of the VF partitioning algorithm.

**Figure 4:**
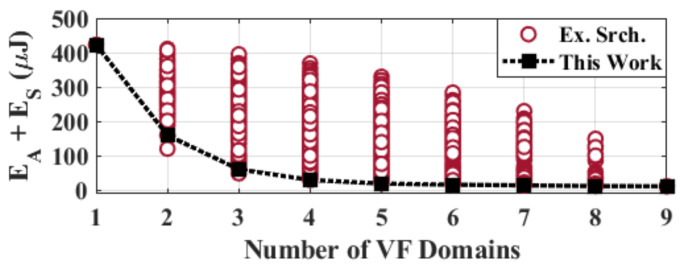
Exhaustive search iterations and the minimum curve captured by the proposed algorithm.

**Design Space Complexity:** Suppose that the total number of always-on and sporadic modules is M=|A|+|S|. The number of ways to partition *M* modules into *K* non-empty sets is given by the Stirling number of the second kind, s(M,K). The sum of all possible partitions of a set, which is $\sum_{K=0}^{M}s\left(M,K\right)$, is the Bell number Bell(M), which grows exponentially [19]. To cope with the complexity, we first consider the always-on and sporadic modules separately; this reduces the design space significantly without ruling out any promising solution since placing sporadic modules into the same domain with always-on modules would undermine their power-gating potential. Subsequently, we present an efficient iterative algorithm, illustrate it using a small problem size in Figure 3 for M=4, and compare its results against an exhaustive search for a larger problem size in Figure 4 for M=9.

**Proposed VF Partitioning Algorithm:** We start by placing each module into a dedicated VF domain, since it can achieve the minimum application energy without considering the VF domain overhead (top row in Figure 3). Subsequently, the proposed algorithm builds its way down to a single domain by iteratively merging VF domains. While moving from *M* to M−1 domains, it explores all possible combinations, i.e., M2 similar to the exhaustive search, as shown in Figure 3 (six partitions with three domains). It applies the optimization technique presented in Section 4.1 for each of these partitions to find the optimum voltage-frequency values and the minimum possible energy consumption. Subsequently, the proposed iterative algorithm selects the partition that gives the minimum total energy consumption. After this point, the algorithm explores only the partitions that can be obtained starting with the current solution. In Figure 3, for example, it decides that the best three-domain solution is the one that merges modules *a* and *d*, i.e., |a,d|b|c|. Hence, it further explores only |a,b,d|c|, |a,c,d|b|, and |a,d|b,c|, which can be obtained by merging only one additional domain. In general, the proposed algorithm explores M−12 combinations, in contrast to the exhaustive search, which would consider all partitions with M−2 domains (i.e., third row in Figure 3). The algorithm continues merging domains until reaching the single domain solution. Finally, it chooses the configuration with the minimum total energy consumption when considering the VF domain overhead. We stress that the algorithm also includes $E_{overhead}$ when performing the partitioning of the domains using the approach described in Section 5.3.1.

**Complexity of the proposed VF Partitioning Algorithm:** When there are a total of *M* modules, the algorithm explores 1+M2+M−12+...+22 partitions. Hence, its complexity is $O(M^{3})$. Because we apply the algorithm to always-on and sporadic modules separately, the overall computational complexity is ${O(|A|}^{3}{)+O(|S|}^{3})$, which is significantly lower than the Bell number Bell(|A|+|S|).

**Figure 5:**
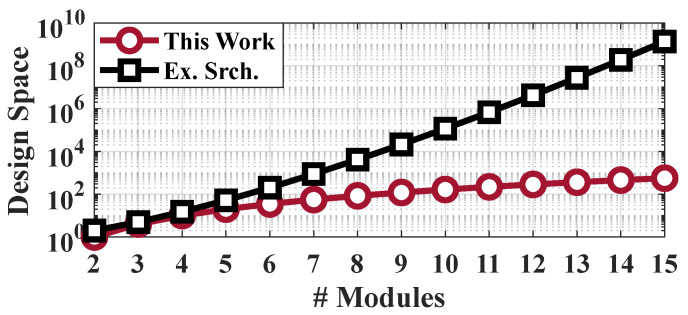
Design space of Exhaustive Search and the Proposed Framework with respect to # of modules.

**Comparison to Exhaustive Search:** We also implement an exact algorithm, which is practical only for small problem sizes, in order to demonstrate the effectiveness of the proposed algorithm. Figure 4 depicts the minimum energy consumption found by the exhaustive search and our algorithm for a synthetic example with nine modules before adding the VF overhead. Each circle marker stands for a different partitioning evaluated by exhaustive search. The proposed algorithm closely follows the minimum energy solution and successfully finds 82% of the optimum solutions by exploring less than 1% of the partitions. As a result, the execution time of our algorithm is about 34 times lower than the exhaustive search. Furthermore, the energy consumption of the other partitions is within 3% of the minimum energy that is achieved by the exhaustive search. The growth in design space for the exhaustive algorithm and the proposed iterative framework is depicted in Figure 5. As can be seen, the design space for exhaustive search grows much more rapidly when compared to the design space of the proposed algorithm.

## 5. Experimental Results

### 5.1. Experimental Setup

We evaluate the proposed framework on three application benchmarks. This section introduces these benchmarks, gives a detailed explanation of the experimental methodology to apply the framework to each of these applications, and illustrates the energy savings obtained by the proposed framework.

#### 5.1.1. Driver Applications

We evaluate the proposed framework on two activity monitoring applications [7], and an ECG application [1]. The activity monitoring applications are developed in-house by implementing them in the Verilog hardware description language. The designs are validated by performing both behavioral and gate-level simulations. For the ECG application, we use the parameters that were reported by the authors.

#### 5.1.2. Experimental Methodology

**Figure 6:**
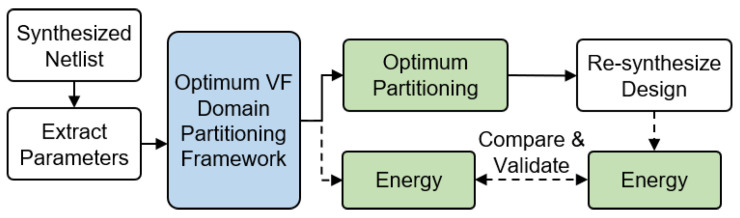
The steps to apply the framework to a design whose design files are available.

**Experimental flow with design files:** When the design files are available, we start by synthesizing the target design using Synopsys Design Compiler and TSMC 65 nm LP standard cell library, as depicted in Figure 6. Subsequently, Synopsys PrimeTime is used to extract the design parameters, such as leakage current, activity factor, capacitance, and the number of active cycles of each module. We report the parameters of the activity monitoring applications in the upcoming Table 2 and Table 3 in order to enable reproducibility of the results. The TSMC library that we use has standard cells defined for 1 V and 1.2 V. We characterized the minimum required supply voltage as a linear function of the frequency as $V\left(f\right)=2\times 10^{-8}f+0.9$ for 0<f≤30 MHz. The proposed framework uses these inputs and relation to generate the optimum VF domain partition, voltage-frequency pair of each domain, and the estimated total energy consumption. Subsequently, we implement the optimum VF domain partition and find its energy consumption. Finally, we validate our results by comparing the actual energy consumption against the estimate that was reported by the proposed framework. This experimental methodology is used for the two activity monitoring applications.

**Experimental flow without design files:** When design files for an application are not available, we use system-level characterizations to estimate the activity factors and processing times of the modules. The proposed optimization framework uses these parameters to determine the optimum VF partitioning of the application. Finally, we compare it against the baseline measurements and previous approaches for VF partitioning. We use this methodology for the ECG application.

### 5.2. Activity Monitoring Application

We start with a detailed analysis of the in-house activity monitoring applications, since we can obtain fine-grained information about them. The two activity monitoring applications share the same always-on modules for sampling and filtering but have disparate sporadic modules that are responsible for the classification of activities. In the following, we provide brief descriptions and information regarding these designs. Much more detailed explanations about these are available in our previously published work [7]. For both designs, we use an activity window of one second, which is representative of human activities [5].

#### 5.2.1. Single-Level Activity Monitoring Design

There are five always-on modules (A1–A5) and four sporadic modules (S1–S4) in this design. The sporadic modules first compute fast Fourier transform, wavelet transform, and statistical features. Afterwards, they use a neural network with a single hidden layer to classify the activity. The key design parameters for always-on and sporadic modules are summarized in Table 2 and Table 3a, respectively. We also implemented a baseline design with one always-on domain and one sporadic domain. This baseline design is manually optimized to meet the sampling and activity window deadlines. The energy consumption of the always-on and sporadic domains over a one second activity window is found as 2.6 μJ and 94.5 nJ, respectively, at 10 kHz operating frequency.

#### 5.2.2. Hierarchical Activity Monitoring Design

The proposed framework is also applied to a larger design with eight sporadic modules. Unlike the earlier single-level design, this hierarchical design invokes a different classifier that is based on the complexity of the input activity; it first categorizes the input activity as simple or complex. Subsequently, it employs a decision tree for simple activities and a neural network for complex activities. This architecture allows for more power gating opportunities. The parameters of this design are summarized in Table 3b. Similar to the first design, we implemented a baseline with one always-on domain and one sporadic domain, both running at 10 kHz. The energy consumption of always-on and sporadic domains over one activity window is found as 2.6 μJ and 91.7 nJ, respectively.

### 5.3. Optimization Results

#### 5.3.1. Energy Overhead of VF Domains

New voltage domains introduce level shifters, sleep transistors, isolation cells, retention cells, and changes in voltage regulators, which lead to area, delay, and energy consumption overhead. Precise number and placement of these components, hence the amount of overhead, is a function of the target design. For our designs, the area overhead is less than 10%, while the delay overhead is negligible. We account for two types of energy consumption overheads, which is more critical for our target applications. First, we consider the energy overhead that is proportional to the number of VF domains [20]. This covers the energy consumption of additional level shifters, isolation and retention cells. The energy consumption overhead of a single level shifter is in the order of fJ [21]. Using the worst-case values, the overhead of additional logic per new domain is estimated as 0.2% in our designs. Second, we consider the total energy consumed by the power gating logic as 5% following the guidelines in [22]. The energy consumption overhead due to voltage regulators is estimated to be less than 1% [23]. Using these values, we estimate the energy consumption overhead of each additional always-on domain as 2.6μJ ×6%=0.15μJ. Similarly, each new sporadic domain incurs 5.67 nJ and 5.50 nJ energy for the single-level and hierarchical designs, respectively.

#### 5.3.2. Single-Level Activity Monitoring Design

The minimum energy configuration for the single-level design has three always-on VF domains with the following partition: |A2,A3|A1,A4|A5|. The proposed framework finds the energy-optimal frequencies of these domains as |1|3|10| kHz. The analysis presented in Section 4.1 suggests grouping the always-on modules with the same minimum frequency would yield the minimum energy. These results show that this is indeed the case. As a result, the energy consumption of the always-on modules ($E_{A}$) reduces by 45% from 2.60 μJ to 1.45 μJ.

**Figure 7:**
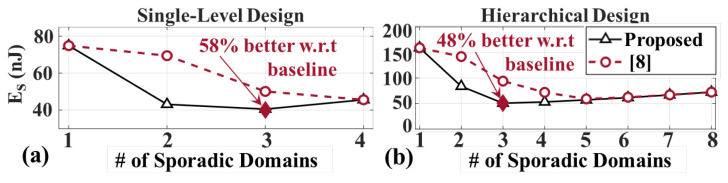
$E_{S}$ vs. # of sporadic domains for designs in (**a**) Section 5.2.1 (**b**) Section 5.2.2.

Figure 7a illustrates the energy savings achieved by the proposed framework in the sporadic domain. With one sporadic domain, the framework finds the energy optimal frequency as 4.35 MHz, and reduces the energy consumption of the sporadic modules by 16%, from 94.5 nJ to 80 nJ. The minimum energy is achieved when there are three sporadic domains. The optimum partitioning of modules into three domains is found as: |S2|S1,S4|S3| with frequencies |3.96|4.48|4.90| MHz. This configuration reduces the energy consumption of sporadic domains ($E_{S}$) by 58%, from 94.5 nJ to 40.0 nJ. Furthermore, we achieve 12% savings when compared to the VF partitioning approach in [8].

#### 5.3.3. Hierarchical Activity Monitoring Design

The results for always-on modules are the same as the previous design, since the modules are common. The minimum energy consumption of the sporadic domains is plotted as a function of number of VF domains in Figure 7b. With one sporadic domain, the energy consumption of the sporadic modules is found as 150 nJ @ 4.11 MHz, which is higher than the 91.7 nJ @ 10 kHz figure reported in Section 5.2.2. This is because 91.7 nJ is the post placement & route value from a testbench that covers both simple and complex type of activities, whereas the 150 nJ reported by our algorithm in Figure 7b only considers complex activities, i.e., only neural network as the classifier. Therefore, the results presented in Figure 7b are pessimistic yet correct. Furthermore, this figure shows that the optimum number of sporadic domains is three. The framework yields the optimum VF domain partitioning and frequencies as: |S1,S5,S7|S3,S4,S6|S2,S8| and 4.01|4.10|4.18 MHz. The energy consumption of sporadic domains with this configuration becomes 49.6 nJ. As a result, the proposed framework achieves approximately 45% and 48% energy savings when compared to the baseline for always-on and sporadic domains, respectively. Similarly, we achieve 16% savings when compared to the state-of-the-art approach in [8].

#### 5.3.4. ECG Application Validation

The baseline energy consumption values for the ECG application are extracted from [1]. According to these, always-on energy consumption is 17.3 μJ, while sporadic energy consumption is 2.6 μJ. Similar to activity monitoring applications, we estimate the energy overhead per domain from these values as 1.07 μJ and 0.16 μJ, respectively. The proposed framework reduces the energy consumption of the design to 11.3 μJ and 1.72 μJ, resulting in 35% and 33% savings for always-on and sporadic energy consumption, respectively. These values are optimal when compared to the exhaustive solution. Additionally, our proposed solution and the solution in [8] achieve the same savings.

### 5.4. Validation of the Framework

We implemented the optimal VF domain partitions reported by the proposed framework in order to validate the energy consumption results. Subsequently, we used Synopsys PrimeTime to obtain the energy consumption of these designs.

**Single-Level Activity Monitoring Design:** After implementing the optimal configuration, the energy consumption of always-on and sporadic domains is found as 1.47 μJ and 36.0 nJ, respectively. By contrast, the proposed framework estimated these values as 1.45 μJ and 40.0 nJ, respectively. This shows that the estimated always-on energy by the framework is within 2%, and the estimated sporadic energy is within 10% of their corresponding actual post-synthesis values. Hence, we indeed achieve close to 45% and 58% reduction in always-on and sporadic energy, respectively, as reported by the proposed framework.

**Hierarchical Activity Monitoring Design:** Always-on energy for this design is the same as the first design (1.47 μJ), as expected, while the sporadic energy consumption is 49.0 nJ. The framework outputs (1.45 μJ and 49.60 nJ) again agree with these results. The estimated sporadic energy is within 2% of the actual post-synthesis value. Therefore, for the given design, we obtain close to 45% and 48% reduction in always-on and sporadic energy, respectively.

### 5.5. Discussion of the Results

We showed that the minimum energy consumption that was reported by the proposed framework matches closely with the energy consumption of the actual design. Our framework is able to predict the energy consumption of different VF domain partitions accurately. Consequently, it accelerates design space exploration immensely by reducing the number of implementations and re-synthesis of the design. The execution times of the three algorithms are shown in Table 4. In addition to these, exhaustive search and our iterative algorithm take 46.8 s and 1.3 s respectively, for the arbitrary design with M=9 modules used in Figure 4. When considering these results and the exponential growth of the design space with number of modules, an exhaustive search for a design with M=15 modules takes in the order of days to execute. Our proposed framework reduces this execution time to hours. Furthermore, the optimum VF domain partitions found by the proposed framework resulted in a total energy consumption of 1.5 μJ. This energy budget is within the energy harvested by a 1 cm^2^ solar cell over one second when operating indoors, which suggests that energy-neutrality can be achieved. Finally, we provided numerical values for the parameters of two activity monitoring application implementations. These can be used by other researchers to develop and evaluate other low power optimization techniques.

#### 5.5.1. Wider Applicability of the Proposed Framework

**Separation of Modules:** We argue that every module in a given design can be considered as either an always-on or a sporadic module, and can therefore be included in the framework. For example, the modules that control a colored display may fall under sporadic domain, whereas the display itself may be considered an always-on module. Similarly, depending on the application, we believe it is possible to identify any sensor as always-on or sporadic. For example, a camera may be always-on for a video based application, but can be sporadically accessed for an image based application. We consider sampling modules as always-on modules in this study.

The energy consumption of the benchmark applications in the manuscript are dominated by always-on modules. However, this is not always the case for all applications. Some applications may contain high-power modules in the sporadic domain, such as more complicated classifiers, like CNNs, or communication modules. In such cases, the optimization of sporadic modules is not negligible. The proposed framework will in fact yield higher energy savings when always-on and sporadic domain consumptions are comparable.

**Different Designs:** For each different design, the proposed algorithm needs to rerun. For example, the two activity monitoring examples in the manuscript share the same always-on modules, but different sporadic modules. Therefore, for the always-on domain partitioning, the optimal solution is shared between the two. However, for sporadic domain partitioning, the proposed algorithm needs to be run for each design separately.

**User Profile:** We did not consider the effect of user activity profile in the current manuscript. However, in a system with always-on and sporadic modules, the energy consumption of always-on modules will be independent of whether there is user access or not. On the other hand, sporadic modules turn on only when the user is active. Therefore, if the user is not active, the energy consumption approaches the consumption of the always-on modules, which are needed for monitoring.

## 6. Conclusions

Wearable devices have great potential to reshape healthcare and doctor-patient interaction. The ubiquity of wearable devices is hindered by their higher power consumption than the capacity of energy harvesting solutions. In regards to this, this paper presented a system-level framework in order to leverage application-specific knowledge into low-power design techniques to explore the voltage-frequency domain design space. This exploration is enabled by a novel efficient iterative algorithm. We applied the proposed framework to three designs, and provided experimental results to show the energy savings enabled by the proposed methodology. We obtain a 33–58% improvement in energy savings over a manually optimized baseline design.

## Figures and Tables

**Table 1:** List of major parameters (m∈{A,S}).

Symbol	Description	Symbol	Description
A,S	Set of *always-on* and *sporadic* modules, respectively	$E_{A}$	Total energy of *always-on* modules
$E_{S}$	Total energy of *sporadic* modules	$\alpha_{m}$	Switching factor of module *m*
$C_{m}$	Capacitance of module *m*	*M*	Total no. of modules
$I_{m}$	Leakage current of module *m*	$t_{a}$	Activity window duration
$n_{m}$	Active cycles of module *m*	D	Set of all domains
V(m)	Voltage of module *m*	f(m)	Frequency of module *m*
$t_{exe}$	Exe. time of a module at a given frequency f(m)	$t_{max}$	Maximum allowed exe. time of a module *m*
$f_{min}\left(m\right)$	Minimum frequency of module *m*	$f_{opt}\left(m\right)$	Optimum frequency for module *m*

**Table 2:** Always-on module parameters of the activity monitoring applications.

	A1	A2	A3	A4	A5
I (nA)	80	100	1000	10	50
αC (pF)	3.41	11.20	89.30	1.02	3.55
$f^{min}$ (kHz)	3	1	1	3	10

**Table 3:** Sporadic module parameters of the (**a**) single-level (**b**) hierarchical activity monitoring designs.

(**a**)				
	**S1**	**S2**	**S3**	**S4**
I (nA)	71	1000	1000	700
αC (pF)	5	100	43	35
Cycles	109	163	25	252

(**b**)								
	**S1**	**S2**	**S3**	**S4**	**S5**	**S6**	**S7**	**S8**
I (nA)	722	755	417	182	36	2670	123	866
αC (pF)	58	29	41	7	5	193	9	81
Cycles	252	25	4	5	252	21	109	145

**Table 4:** Execution time of the three algorithms on three benchmark designs. The numbers in parentheses show the number of modules in that design.

	ECG (2)	Single-Level Design (4)	Hierarchical Design (8)
Exhaustive Search	15 ms	54 ms	7.70 s
[8]	0.20 ms	10 ms	0.05 s
This Work	0.01 ms	8 ms	0.20 s
